# Significance of Combined Analysis of High-Risk Human Papillomaviruses Polymerized Chain Reaction Analysis and Immunohistochemical Expression of p16INK4A in Cervical Cancer in a Cohort of South-Indian Population

**DOI:** 10.7759/cureus.29001

**Published:** 2022-09-10

**Authors:** Thathapudi Sujatha, Surekha Mullapudi Venkata, Erukkambattu Jayashankar, Uday Kumar Putcha, Sandeep Kumar Koturu, Triveni Bhopal, Krishnaveni Neelala, Sanjeeva Kumari Chinta, Reji Manjunathan

**Affiliations:** 1 Department of Pathology and Microbiology, National Institute of Nutrition, Tarnaka, Hyderabad, IND; 2 Department of Pathology and Laboratory Medicine, All India Institute of Medical Sciences, Bhopal, Bhopal, IND; 3 Department of Molecular Biology, National Institute of Nutrition, Tarnaka, Hyderabad, IND; 4 Department of Pathology, Mehdi Nawaj Jung (MNJ) Cancer Hospital, Hyderabad, IND; 5 Department of Pathology, Central Railway Hospital, Secunderabad, Hyderabad, IND; 6 Department of Medical Oncology, Mehdi Nawaj Jung (MNJ) Cancer Hospital, Hyderabad, Hyderabad, IND; 7 Medical Records Unit, Chengalpattu Government Medical College, Chengalpattu, IND

**Keywords:** hr-hpv, cervical cancer, squamous intraepithelial lesions, p16ink4a, human papillomavirus (hpv)

## Abstract

Introduction

Cervical cancer is the fourth most frequent cancer in women worldwide, and it continues to be a big issue in developing countries. The current case-control study sought to determine the presence of high-risk human papillomaviruses (hr-HPV) in the development of cervical cancer, as well as their relationship with the cell cycle inhibitor gene p16INK4A in cervical cancer.

Methods

The association between p16INK4A protein and the presence of hr-HPV DNA in cervical lesions was explored in this study, which included 150 cervical cancer patients and 100 normal cervix samples. The immunohistochemistry approach was used to identify the expression of the p16INK4A protein, while the semi-quantitative polymerized chain reaction (PCR) method was used to identify the genomic identity of hr-HPV.

Results

About 90.67% (n=136) of the 150 case samples were found to be hr-HPV positive. Within the 136 HPV-positive samples, 45 (33.08%) show moderate expression of the p16INK4A protein, whereas 91 (66.91%) show overexpression, which is statistically significant (0.05). Among the 136 HPV-positive samples, 22.08% (N=30) were classified as having cervical intraepithelial neoplasia (CIN), with 56.66% (n=17) having CIN3, 36.66% (n=11) having CIN2, and 6.67% (n=2) having CIN1.

Conclusion

Based on the semi-quantitative immune staining scoring method of p16INK4A protein, genomic expression of HPV demonstrates that the expression of p16INK4A protein increases with the infectious load of the hr-HPV genome in the host cell. The result directly shows that immunostaining of the p16INK4A protein, in conjunction with the assessment of high-risk HPV in the host genome, will aid in the identification of cervical cancer in the cervix.

## Introduction

Uterine cervical cancer remains the most common cause of death for women, ranks fourth among all cancers worldwide in females, and is the first major human cancer reported with a single infectious etiology [1]. It is the second most common cancer form among Indian women and represents 16.5% of the total cancer cases reported so far. Based on the available data, it is estimated that in India, almost 160 million women between the ages of 30 and 59 years are at high risk of developing cervical cancer [2]. Infection due to human papillomaviruses (HPV) is identified as the major causative reason behind cervical cancer [3]. Among the identified types of HPV, the two types HPV16 and 18 cause 82.5% of cervical cancer and pre-cancerous cervical lesions [4]. Genomic integration of the HPV viral genome can disrupt the regulation of several cervical epithelium cellular proteins. The E6 and E7 oncoproteins from HPV have been shown to bind with p53 and retinoblastoma proteins and result in dysregulated cell death and the release of cell proliferation transcription factor E2F. Overexpression of E2F inhibits cyclin D1-dependent kinase (CDK) activity and induces the expression of p16INK4a transcript [5,6]. Elevated expression of p16INK4a can slow down the cell cycle progression by inactivating the CDK that phosphorylates the retinoblastoma protein and can accelerate the immortalization process among HPV-infected neoplastic cervical epithelial cells [7]. Hence, the identification of p16INK4a overexpression is used as a biomarker of malignant transformation by HPV in neoplastic cervical epithelial cells during the diagnosis of cervical cancer.

Biomarker-based test systems have been developed and clinically evaluated on the basis of a clarified understanding of the molecular pathogenesis of how HPVs contribute to the neoplastic transformation of cervical squamous epithelial cells [8]. In particular, the identification of p16INK4a as a marker for “transforming” HPV infections added more accuracy to early cancer detection and diagnostic programs [9]. Many reports from India, found that immunostaining of the protein p16INK4a is a convincing method to identify the high-risk progression of cervical cancer malignancies with great specificity and thereby necessitate future treatment [10]. Gupta et al. (2010) showed an excellent correlation of p16INK4a expression for the grading of cervical intraepithelial neoplasia (CIN) and identified it as a sensitive marker of CIN [11].

Though previous studies mentioned that immunohistochemical (IHC) identification of p16INK4a could be a biomarker to predict the outcomes of cervical cancer lesions, the predominance of comparing as a biomarker along with HPV genotyping was not fully elucidated. Therefore, in the present study, we aimed to identify the existence of the HPV genome along with the immunoexpression of p16INK4a protein in the uterine cervix as an effective biomarker to identify the role of high-risk HPV in the development of cervical cancer and also to find an association between the HPV genome and the cell cycle inhibitor protein p16INK4a. Doing so, we could provide a strong inference from India to strengthen the association between p16INK4a and HPV for the early detection of high-grade cervical lesions and cervical cancer tissue toward better prophylaxis of the scenario.

## Materials and methods

Study population and specimen collection

The Institutional Ethics Committee, MNJ Institute of Oncology and Regional Cancer Center, Hyderabad, India, reviewed and approved the protocol on July 12th, 2018 (Regd No. ECR/227/Inst/AP/2013/RR-16). The prospective study consists of 250 samples, 150 cases and 100 controls. A total of 150 histopathologically diagnosed specimens were included for cervical tumors. Both the cervical samples and normal tissues were obtained in normal saline and formalin and were processed based on the methodology demands. Cervical biopsy positive for dysplasia or malignancy in epithelial cells was included, and the sections that were negative for dysplasia or malignancy were excluded from the study.

Histopathological analysis

Sample biopsies were fixed in 10% neutral buffered formalin, dehydrated and paraffin-embedded. Formalin-fixed paraffin-embedded blocks were sectioned on a microtome (5 μm in thickness) for both hematoxylin and eosin (H&E) staining and immunostaining. The H&E stained slides were examined for histopathological diagnosis by an experienced pathologist to record morphological alterations [8].

Immunohistochemical staining and semi-quantitative scoring

All the cervix carcinoma samples were diagnosed histologically and were subjected to immunohistochemical analysis for p16INK4A expression as described elsewhere [12]. The antibody titer and staining parameters for the protein p16INK4A were optimized on the recommended control tissue according to the manufacturer’s instructions (Dako, Glostrup, Denmark). In brief, the fixed tissue sections of 5 μm in thickness were blocked and incubated with p16INK4A primary antibody for 20 minutes. After the washing procedure, the sections were incubated with a secondary antibody coupled with horse radish peroxidase (HRP). The reaction was developed using 3,3′-Diaminobenzidine (DAB) Chromogen (Dako, Glostrup, Denmark) and after dehydration, the sections were counterstained with hematoxylin stain. 

Semi-quantitative scoring of immunostained cervical cancer sections was performed by an experienced pathologist. Normal cervical tissue blocks were used as controls. Strong, diffuse nuclear, as well as cytoplasmic staining in the tumor cells of the squamous epithelium, is considered p16INK4A positive. First, the points were given based on the intensity of staining and as follows: a grade of 0 is given for no staining; 1 for weak staining; 2 for moderate staining; and 3 for strong staining. Secondly, the points were given according to the proportion of cells stained for p16INK4A as follows: 0 is given for no staining; 1 for <1%; 2 for 1-10%; 3 for 11-33%; 4 for 34-66%; and 5 for >66% of cell staining. The final score was tabulated by adding the points of intensity and the proportion of cells stained for the p16INK4A primary antibody. The maximum score was given 8 and the minimum of 0. A score of 0 to 2 was taken as low expression, in-between 3 and 5 as moderate expression, and a score calculated in-between 6 and 8 was taken as overexpression [2,12].

Identification of HPV viral genome expression by semi-quantitative PCR method

The procedure for the identification of high-risk HPV viral genome expression from fresh tissue samples was followed as described elsewhere [13]. In brief, genomic DNA was extracted from fresh tissue (case) and from archival tissues (control) using the respective DNA mini kit isolation kit (Genetix) as per the manufacturer's instruction. The extraction quality and quantity were analyzed using a Nanodrop (ND1000, BIORAD). HPV detection was made by the polymerized chain reaction (PCR) method using the primer set for high-risk HPV for region E6. The primer sequences for the product of band size 630 base pairs are as follows: HPV 3f: GGG WGK KAC TGA AAT CGGT; HPV 6b (r): TCC TCT GAG TYG YCT AAT TGC TC; HPV 5b (r): CTG AGC TGT CAR NTA ATT GCT CA [14] and were obtained from euro fins. Amplification reactions contained a final volume of 50 µL and were performed with 18 µL of Takara PCR master mix kit (Takara, Takara Bio Inc), 0.5 µM concentrations of each primer, and 1 µL of DNA samples. PCR was performed based on the following cycling conditions: first, three minutes at 95°C; then 40 cycles of 95°C for 15 s, 60°C for 20 s, and 72°C for 25 s; with a final elongation of seven minutes at 72°C using the CFX96 (BioRad) machine. PCR products of 5 µL in volume were electrophoresed in a 2% agarose gel (Agarose; Roche). Images were recorded using a Geldoc 2000-System (BioRad).

Statistical analysis

Statistical analysis was performed using SPSS version 20.0 (IBM SPSS Statistics for Windows, Version 20.0., IBM Corp., Armonk, NY). A descriptive statistics test was used to determine the statistical significance. The p-value of <0.05 was considered statistically significant.

## Results

**Figure 1:**
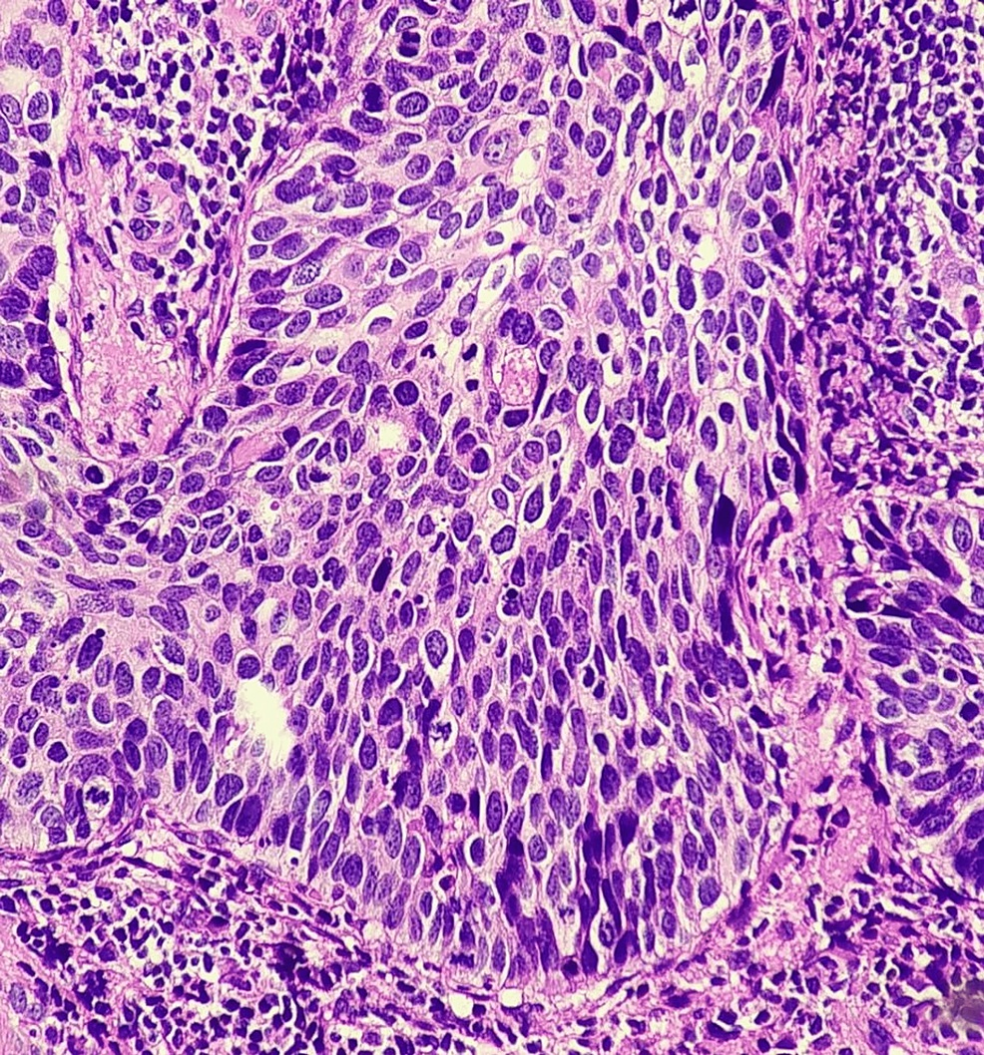
H&E image. Squamous cell carcinoma of the cervix, on high power 400× hematoxylin and eosin section.

**Figure 2:**
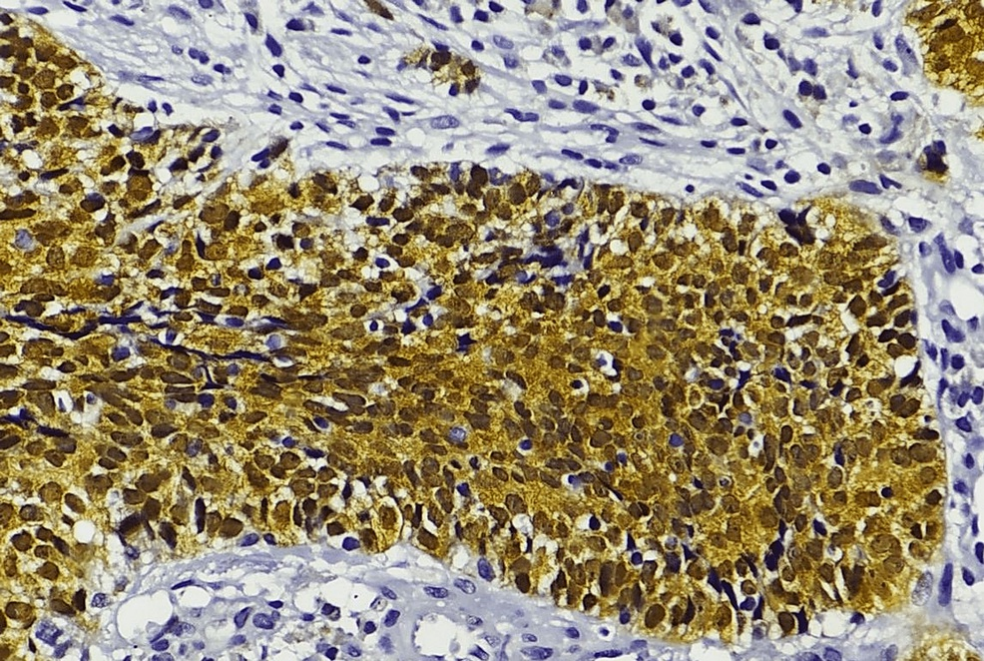
Immunohistochemical image of p16INK4A. Strong nuclear and cytoplasmic positivity of p16, on high power, 400×.

**Figure 3:**
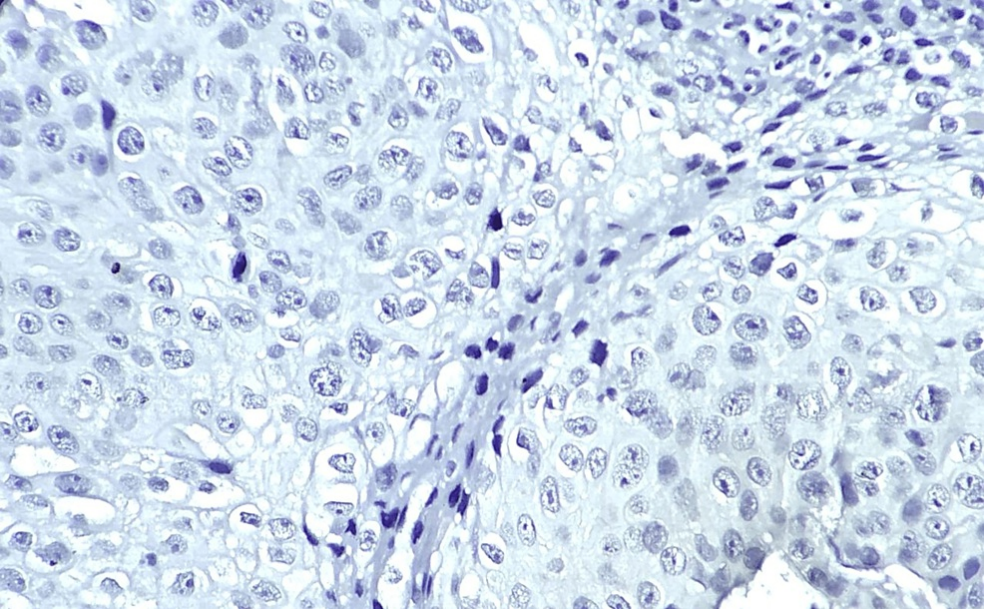
p16 IHC negative image. p16 IHC-negative on high power, 400×. IHC: immunohistochemical.

This study includes 250 samples, of which 150 were reported with squamous cell carcinoma (cases) and 100 without squamous cell carcinoma (controls). The expression of the high-risk HPV genome was analyzed using the semi-quantitative PCR method. The data show that among 150 case samples, 90.67% (n=136) of the cases were identified with the presence of the HPV genome and are categorized as HPV-positive samples. Only 9.33% (n=14) were found to be negative for high-risk HPV in their host genome. Hematoxylin and eosin staining was performed to identify the changes in the cellular morphology (Figure 1). Immunostaining expression and semi-quantitative scoring of p16INK4A protein were performed among samples (Figures 2, 3). The data indicate that among the 150 case samples, the samples that are positive for the HPV genome (n=136) show the expression of the p16INK4A protein. Samples that are grouped under the category of negative for high-risk HPV in their host genome expression (n=14) don’t show the presence of p16INK4A protein. The immunostaining intensity of p16INK4A protein among HPV genome-positive samples was calculated and is given in Table 1. The data emphasize that among the 136 positive samples, 45 samples (33.08%) show moderate expression of p16INK4A protein. On the other hand, almost 91 samples (66.91%) from the HPV-positive cases show overexpression of p16INK4A protein both in the nucleus as well as in the cytoplasm of squamous epithelial cells and is statistically significant (<0.05). The same group was also noted with many dysplastic cells.

**Table 1:** Scoring of p16 Immunohistochemical stain. The table represents a moderate expression of 33% and overexpression of 67% of p16 immunohistochemical staining in a total of 136/150 cases.

Immunohistochemical expression of p16 INK4A marker					
Moderate expression			Overexpression		
Score	No	% of Population	Score	No	% of Population
3	06	2.2	6	25	18.38
4	34	25	7	03	2.2
5	05	3.67	8	63	46.32
Total	45	33.08	Total	91	66.91
Significance p is <0.05, moderate versus overexpression					

Among the 136 HPV-positive samples, 22.08% of the individuals (N=30) were grouped under cervical intraepithelial neoplasia (CIN). Within the CIN identified, 56.66% (n=17) were noticed with CIN3, 36.66% (n=11) were with CIN2 and 6.67% (n=2) were with CIN1. We noticed that the two individuals grouped under CIN were found to be negative for HPV but they expressed p16INK4A protein with a low score.

**Figure 4:**
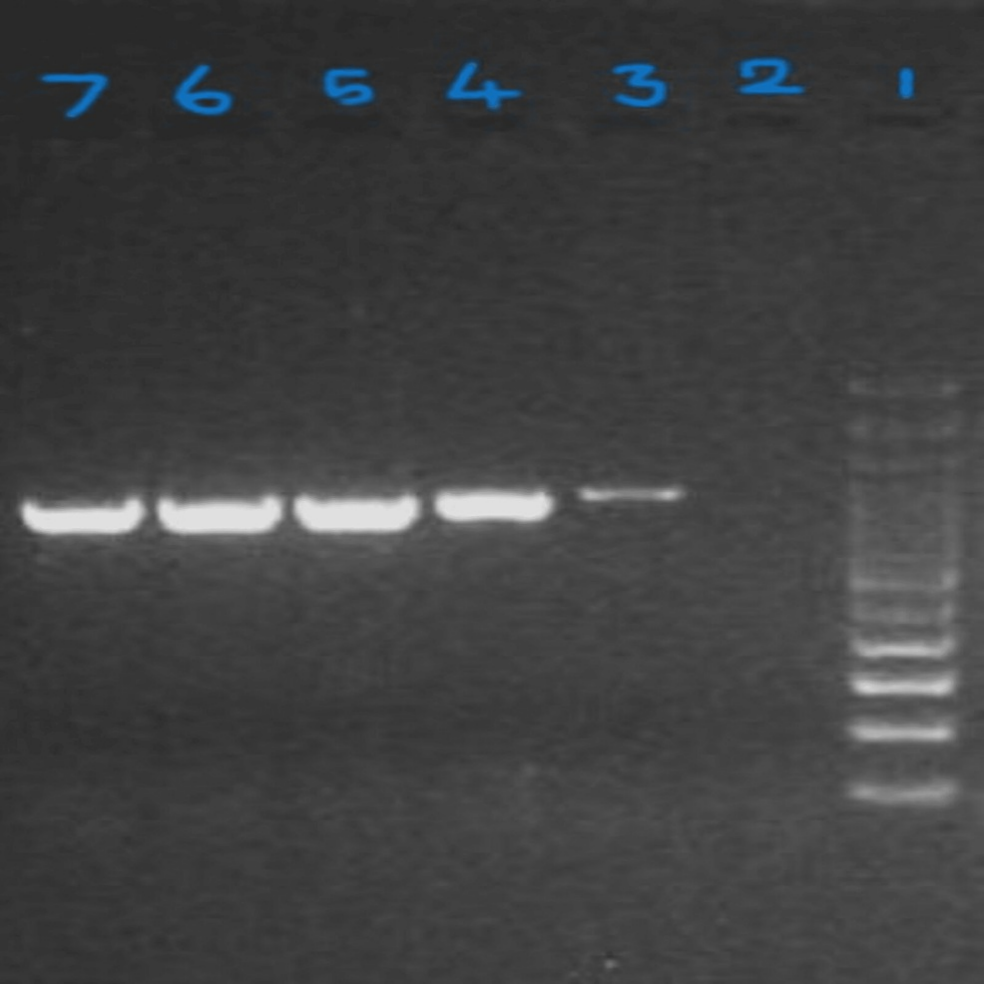
2% Agarose gel picture of HPV in cervix cancer. HPV PCR gel image: lane 1: 50 bp ladder, lane 2: negative control, lane 3: moderate expression of HPV DNA at 630 bp region, and lanes 4, 5, 6, 7: strong expression of HPV DNA at 630 bp region. HPV: human papillomaviruses, PCR: polymerized chain reaction.

Categorization of high-risk HPV genome expression was performed based on the immunostaining scoring of the p16INK4A protein. Figure 4 represents an agarose gel image of the high-risk-HPV genome (based on the scoring pattern) among the p16INK4A protein-positive samples. The data show that the intensity of the PCR products increased directly in proportion to the staining score of p16INK4A protein in the tissues. Thus, the result directly indicates that in cervical squamous carcinoma cells, expression of p16INK4A was found to intensify based on the viral genome load of the high-risk HPV.

## Discussion

HPV is a definite etiological agent and a sensitive marker for the identification of the risk of progression of cervical neoplasia in cervical cancer patients [1]. The tumor suppressor gene p16INK4A is now widely accepted as a sensitive and specific marker for the diagnosis of cervical squamous dysplastic cells and is a valuable addition to cervical squamous cancer screening [8]. Though the prognostic value of overexpression of p16ink4A protein in cervical cancer has been evaluated for several years, the result remains controversial [14]. However, only a few data points are available from India to strengthen the association between p16ink4A and the presence of HPV in high-grade cervical lesions [15]. In this particular study, we tried to analyze the association of p16ink4A protein through immune expression along with the identification of HPV, especially the high-risk HPV genome in the host cell.

Cervical carcinogenesis is a complex mechanism due to the integration of the HPV gene into the host cell transcription machinery, which results in the destruction of the immune response of host cells [16]. The role of HPV in cervical carcinogenesis has been well defined and is found to be associated with the activation of the viral oncogenes E6 and E7 in the basal and para-basal cells of the infected epithelium [17]. In this particular study, we analyzed the expression of the high-risk HPV gene in the host genome. Among the analyzed case samples, 136 (90.67%) samples were identified with the presence of the HPV gene in the host cell. The data emphasize that in all reported cervical cancer the expression of the HPV gene is persistent and is considered the causative reason for the propagation of high-grade cervical intraepithelial neoplasia. As a supportive factor, we could find cervical intraepithelial neoplasia (CIN) among HPV-positive cancer samples with more identification under the CIN3 and CIN2 categories (56.66% and 36.66%, respectively).

The immunohistochemical identification of p16INK4A protein in cervical intraepithelial neoplasia has been reported to be a useful diagnostic biomarker for the identification of cervical cancer among women [18,19]. Some reports identify that the rate of expression of p16INK4A protein changes based on the grading of cervical cancer [12,20]. In our study, we found that patients who were positive for the presence of high-risk HPV in their genome noticed the expression of p16INK4A protein in the nucleus as well as in the cytoplasm of squamous epithelial cells. Almost 66.91% of the samples overexpressed the presence of the p16INK4A protein. The data speculated the fact that the expression of p16INK4A protein varies with the infectious potential of HPV with the host genome and is highly associated with the presence of high-risk HPV genome integration with the host cells. This observation is in accordance with previous data, which reported that the semi-quantitative scoring of p16INK4A protein in carcinoma cervix clarified the overexpression pattern of p16INK4A protein in the host cell. 

Limitation of the study

Due to financial constrictions and the limited duration of the study, the sample size is less, and more sample study is required to be more authentic. We have done an hr-HPV PCR study representing combined genotypes of hr-HPV. We have not used specific genotype markers like HPV 16, 18, or 33 separately.

## Conclusions

We conclude that there is a strong association between genome expression of high-risk HPV and immune expression of p16, both in cervical intraepithelial lesions as well as cervical malignancies, highlighting the significance of immunohistochemistry with p16 in cases of cervical cancer, and making p16, a surrogate marker for HPV in low resource settings.
